# Determinants of exclusive breastfeeding: a study of two sub-districts in the Atwima Nwabiagya District of Ghana

**DOI:** 10.11604/pamj.2015.22.248.6904

**Published:** 2015-11-17

**Authors:** Alice Ayawine, Kenneth Ayuurebobi Ae-Ngibise

**Affiliations:** 1 Faculty of Public Health and Allied Sciences, Catholic University College of Ghana, Fiapre, Box 363, Sunyani, Ghana; 2 Kintampo Health Research Centre, Ghana Health Service, Box 200, Kintampo, Ghana

**Keywords:** Exclusive breastfeeding, Atwima Nwabiagya, mothers

## Abstract

**Introduction:**

Optimal breastfeeding rates have not been encouraging globally with sub-optimal feeding being customized in Sub-Saharan Africa. However, in the Atwima Nwabiagya district of Ghana, the message of Exclusive Breastfeeding (EBF) has caught up well with many nursing mothers. we examined the determinants of EBF vis-à-vis performance of a community based growth promotion strategy in the Atwima Nwabiagya district of the Ashanti region of Ghana.

**Methods:**

The study employed a cross-sectional comparative study design to analyze the impact of a community based growth promotion strategy on exclusive breast feeding in Abuakwa and Barekese, both in the Atwima Nwabiagya district of Ghana. Simple random sampling was used to select three communities each from the two sub-districts. Data collection tool employed was a standard questionnaire consisting of closed-ended questions. The variables were EBF knowledge level of mothers, cultural practices affecting EBF practice, occupational hindrances and the level of community participation in EBF activities.

**Results:**

In all three hundred (300) nursing mothers of babies (0-12 months) were purposively interviewed. Results showed that mother's level of knowledge about EBF was good as such the practice was high. In addition, cultural practices in the area did not deter mothers from practicing exclusive breastfeeding. Two factors were associated with EBF in the univariate logistic model. Unmarried mothers were less likely to practice EB compared with mothers who were married (OR = 0.46, 95% 0.28, 0.77). Also the duration of breast feeding was associated wit EBF. The adjusted odds ratio was 0.41(95% CI: 0.32, 0.54) in favor of three months compared with six months.

**Conclusion:**

The Community Based Growth Promotion strategy has had a positive impact on the practice of EBF in the district. It is recommended that the collapsed initiative be reawaken if the stakes are to be maintained. There is also the need to address mothers’ occupational needs and effective breastfeeding practices as this emerged as a major set-back to the practice of EBF among the participants.

## Introduction

According to the World Health Organization (WHO) and United Nations Children and Educational Fund (UNICEF), about 1.5 million babies die every year because they were not breastfed. Several others suffer from contagious diseases and malnutrition, leading to their premature death because they were bottle-fed [1]. Optimal breastfeeding rates have not been encouraging the world over with sub-optimal feeding being customized in Sub-Saharan Africa. The incidence of breastfeeding initiation exceeds 90% in almost every country and is widespread in Sub-Sahara Africa [2]. However, Exclusive Breastfeeding (EBF) during the early stages of the infant's life which is a recommendation from WHO remains critical to the survival of the infant. Breastfeeding is universally practiced; nonetheless, the level of knowledge of mothers and their belief systems regarding exclusive breastfeeding are not well documented [3].

In Africa, more than 95% of infants are breastfed but the feeding practices are often inadequate, feeding water and other liquids to breastfeed infants is prevalent [4, 5]. Prolonged breastfeeding, however, is common and the median duration of breastfeeding ranges between 16 and 28 months. Urbanization and mother's education are cited as the major factors that tend to shorten breastfeeding [6]. The situation in Ghana is not different. Here, women breastfeed for a median duration of 22 months with 53.4% breastfeeding their young babies. The early introduction of additional foods and liquids causes higher rates of diarrhea illness and mortality [7].

To help curb the trend, the concept of EBF was conceived and launched in 1990, through the collective efforts of the World Health Organization and United Nations Children and Educational Fund. The two bodies adopted the Innocentile Declaration on the Protection, Promotion and Support of Breastfeeding. The Declaration recognized that breastfeeding is a unique process which affords infants the idyllic combination of nutrients that provide critical protection against infectious diseases. The protective quality of breast milk decreases the rate of infant morbidity and mortality as well as contributes to the health and wellbeing of children. EBF for six months is the global goal for optimal maternal and child health and nutrition. All women should be encouraged to breastfeed and all infants should receive breast milk for up to two years of age or beyond with complementary foods [8].

Thereafter, breastfeeding began to champion the fore as a public health issue. Owing to the fact that both mother and infant who do not breastfeed are at a momentous disadvantage relative to health outcomes, most health organizations and government agencies promote EBF for the first six months of life with continued breastfeeding and appropriate complementary foods thereafter. Healthy people 2010 set goals for increasing the initiation, duration and exclusivity of breastfeeding. These goals include; 75% initiation rates, 50% continuation of any breastfeeding at six months, 25% of any breastfeeding at one year, 60% of EBF at three months 25% EBF through six months [9].

Available data, however, shows that 72.9% of infants have experienced at least some breastfeeding, 59.4% exclusively breastfed at seven days, 38.7% exclusively breastfed at three months and 13.9% exclusively breastfed at six months [10]. These data disclose that EBF rates are trembling downwards, irrespective of the massive campaigns that have gone in favor of its adoption.

In pursuance of the battle against infant mortality, the World Health Organization and the United Nations Children and Educational Fund again developed the Baby Friendly Hospital Initiative which was launched in 1991. The Nkawie District Hospital became a beneficiary of this initiative. In addition, the Ghana Health Service and Basic II with the sponsorship of USAID launched the community based growth promotion strategy (CBGP) in late 2002 with its objective to improve the level of EBF in certain districts in Ghana including the Atwima Nwabiagya district. Growth promoters, were to among others, educate mothers on exclusive breastfeeding, assist dispel misconceptions (cultural practices) associated with breast feeding and also support mothers to overcome difficulties associated with the practice.

However due to the absence of remuneration, coupled with ineffective monitoring, this laudable initiative toppled. This study aims at identifying the impact of the defunct initiative as well as other factors affecting EBF practice in selected peri-urban and rural sub-districts in the Atwima Nwabiagya District of the Ashanti Region of Ghana.

## Methods

### The study area

Atwima Nwabiagya District is one of the Twenty Seven (27) administrative districts in Ashanti Region with Nkawie as the district capital. It has a Population size of about 197,874 and shares common boundaries with the Ahafo Ano South District to the North, Amansie West District to the south, Kumasi Metropolis to the East and Atwima Mponua District. The district boasts of a state owned hospital in its capital and two other privately owned hospitals in settlement areas. In addition, there are seven health centers and five maternity homes, all boosting up healthcare delivery in the district. Major settlements in the district are Abuakwa, Toase, Akropong, Asuofua and Barekese. The district has several of its rural communities occupying hard to reach areas. This makes healthcare to these communities, especially during the rainy season, a herculean task. None-the less, with the health of the child at its heart of activities, as engrossed in the Health Directorate's vision statement as “a district where every child born lives to celebrate his/her fifth birthday”, the Atwima Nwabiagya district is lauded as one of the first to extend healthcare to the doorstep of the people through its home visitation programme. The district hospital has been awarded a baby friendly status and has further benefitted from several international donor support like the USAID and Linkages [11].

### Study design and population

A cross sectional comparative study design was employed to identify the determinants of EBF in the two sub-districts of Abuakwa and Barekese in the Ashanti region of Ghana. Granted that the district records a 100 percent turn-out at child welfare clinic (district mid-year review 2009), the researchers deemed it appropriate to conduct a community based study. The team travelled alongside the outreach team of the health center in the sub-district to the communities where clinics were held. Interviews were conducted with mothers after they had completed their routine at outreach welfare clinics but at a different location. This is to ensure that mothers provided response in a relatively free environment devoid of intimidation or the watchful eyes of health providers. The time frame for the research lasted a little over a year, spanning through June 2009 to September 2010.

Study population comprised nursing mothers selected from each of the six communities within the two sub-districts under study. Primarily, simple random sampling was used to select two sub-districts among the five sub-districts in the Atwima Nwabiagya district. These were Abuakwa, a peri-urban district and Barekese, a predominant rural sub-district. Three communities each from the two sub districts were further randomly selected. A systematic sampling procedure of an interval of three was then used to contact fifty (50) nursing mothers who had turned up at child welfare clinic organized in each community. Before arriving at the interval used, the researchers did the following basic calculation: the average number of households that had at least a baby of 0-12 months old at the time of survey (200) was divided by the average number of mothers that turned up at child welfare clinic in each community (70). In all three hundred (300) nursing mothers made up the sampled population.

### Study procedures

EBF was studied as the dependent variable. The independent variables included mothers’ knowledge level of exclusive breastfeeding, cultural practices in relation to breastfeeding, mother's occupation and exclusive breastfeeding, and community participation in EBF programs. Quantitative data was gathered from respondents during Outreach Clinics conducted at the communities by staff the two main health centers in the sub-districts. Trained Field Assistants, each from the communities, were recruited for data collection. Questionnaires were translated and administered in the local language (Twi). Questionnaires were pre-tested in Sapaase, a peri-urban community, and Boahenkwa II, a rural community. Both within a sister sub-district.

### Statistical analysis

Data were entered and all statistical analyses carried out in STATA Version 10 (StataCorp, College Station, TX, USA). Unadjusted and Adjusted Odd ratios with 95% confidence interval (95% CI) estimated.

### Ethical approvals

Ethical clearance was obtained from the Okomfo Anokye Ethics Committee, Kumasi and that of the Department of Community Health, Kwame Nkrumah University of Science and Technology, Kumasi. In addition, written permission was sought from the regional and district directors of health services within whose jurisdictions the study took place. The purpose of the study was also explained to participants in a language that was comprehensible to them and they had the choice to participate or abstain. Reference codes were used to replace personal identifiers to ensure participant confidentiality and anonymity. Mothers involved in breastfeeding malpractices were identified and advised on the need to practice exclusive breastfeeding.

## Results

In all, three hundred (300) nursing mothers made up the study population with the two sub-districts sharing equal numbers of respondents (Table 1). Among the three hundred (300) respondents who participated in this study, one hundred and fifty (150) belonged to the Abuakwa sub-district and the other half to the Barekese sub-district. Majority of respondents in both districts came from the 21-30 age group with 79(52.67%) and 85(56.675%) coming from Abuakwa and Barekese respectively (Table 1).

**Table 1:** Demographic characteristics of the study participants

	N= 300			
	Abuakwa	Barekese		
Variable	N (%)	N (%)	Total (%)	P- Value
**Age**				P = 0.248
15-20	24 (16.00)	30 (20.00)	54 (18.00)	
21-30	79 (52.67)	85 (56.67)	164 (54.67)	
31-40	45 (30.00)	31 (20.67)	76 (25.33)	
41-50	2 (1.33)	4 (2.67)	6 (2.00)	
**Educational level**				P = 0.001
None	17 (11.33)	37 (24.67)	54 (18.00)	
Primary	17 (11.33)	28 (18.67)	45 (15.00)	
JHS	98 (65.33)	80 (53.33)	178 (59.33)	
SHS	17 (11.33)	5 (3.33)	22 (7.33)	
Tertiary	1 (0.67)	0 (0.00)	1 (0.33)	
**Place of delivery**				P = 0.006
Health Facility Residence of TBA	127 (84.67)	107 (71.33)	234 (78.00)	
At Home	0 (0.00)	4 (2.67)	4 (1.33)	
	23 (15.33)	39 (26.00)	62 (20.67)	
**Husband's Occupation**				P = 0.000
Civil Servant	34(22.67)	20(13.33)	54(18)	
Farming	34(22.67)	74(49.33)	108(36.00)	
Driving	30(20)	27(18.00)	57(19.00)	
Trading	52(34.67)	29(19.33)	81(27.00)	
**Marital Status**				P = 0.489
Married	47(31.33)	56(37.33)	103(34.33)	
Widowed	4(4.67)	5(3.33)	9(3.00)	
Single	99(66.00)	89(59.33)	188(62.67)	
**Religious Background**				P = 0.002
Christian	139(92.67)	118(78.67)	257(85.67)	
Moslem	10(6.67)	27(18.00)	37(12.33)	
Traditionalist	1(0.67)	5(3.33)	6(2.00)	
**Average income per month**				P = 0.000
<GHC50.00	48(32.00)	84(56.00)	132(44.00)	
> GH₵50 but <GHC100.0	52(34.67)	63(42.00)	115(38.33)	
GHC200 & above	50(33.33)	3(2.00)	53(17.67)	
**Items Owned**				P = 0.000
TV set	71(47.33)	28(18.67)	99(33.00)	
Motor	2(1.33)	1(0.67)	3(1.00)	
Car	5(3.33)	7(4.67)	12(4.00)	
Sound system	71(47.33)	114(76.00)	185(61.67)	
Others	1(0.67)	0(0.00)	1(0.33)	

The second highest recruited nursing mothers were those from the 31-40 age groups. Abuakwa had 45(30.00%) and Barekese had 31(20.67%) respondents. There were more teenage mothers in Barekese than Abuakwa as the figures for the 15-20 age group stood at 24(16.005%) and 30(20.00%) for Abuakwa and Barekese respectively. Mothers in the 41-50 age groups were the least: 2(1.33%) for Abuakwa and 4(2.675%) for Barekese. This shows that nursing mothers between the ages of 15-40 had interest in exclusive breastfeeding.

Most nursing mothers who participated in the study had attained Junior High School/Middle school education with the highest number emerging from Abuakwa with 98(65.33%) respondents. Barekese also had 80(53.33%) respondents attaining their Junior High School/Middle School education. Also 17(11.33%) mothers from Abuakwa had attained primary education as against 28(18.67%) from Barekese. Only 5(3.33%) mothers from Barekese had attained their Senior High School education whereas Abuakwa had 17(11.33%) mothers with the same qualification. Surprisingly, in the sample, Barekese had no mother with tertiary education whereas Abuakwa had only one mother (0.67%). There were more illiterate mothers in Barekese than in Abuakwa. The figures show 37(24.67%) mothers in Barekese had never been to school. This was as against 17(11.33%) from Abuakwa.

Majority of the respondents gave birth at a health center: 114(76.00%) and 102(68.00%) respondents from Abuakwa and Barekese respectively. Only 5(3.33%) mothers in Barekese gave birth in a maternity home whereas 13(8.67%) mothers from Abuakwa also did same. However, more mothers in Barekese 39(26.00%) gave birth at home as compared to 23(15.33%) in Abuakwa. No mother in Abuakwa gave birth at the residence of a TBA whereas in Barekese 4(2.67%) mothers gave birth at the residence of a TBA.

Farming was the major occupation among husbands in the two areas though Barekese recorded higher figures than Abuakwa. Terrifyingly, majority of respondents in both sub-districts were not married. Single mothers recorded 99(66.00%) and 89(59.33%) for Abuakwa and Barekese respectively. Only 47(31.33%) mothers were married in Abuakwa whereas 56(37.33%) were married in Barekese. Also 4 (4.67%) mothers in Abuakwa and 5 (3.33%) in Barekese were divorced.

Most of the mothers interviewed both in Abuakwa and Barekese were Christians. Abuakwa registered 139(92.67%) whilst Barekese registered 118(78.67%). Few mothers earned about GH¢200.00 and above in the two districts. This implies that more mothers in Barekese earned less than GH¢50.00 than in Abuakwa. For those who earned less than GH¢100 a month, the figures recorded 52(34.67%) and 63(42.00%) for Abuakwa and Barekese respectively.

The commonest item owned by most respondents and their partners was sound system with more mothers owning more sound systems in Barekese than in Abuakwa. The results show 71(47.33%) and 114(76.00%) for Abuakwa and Barekese respectively. However, 71(47.33%) respondents in Abuakwa had TV sets as against 28(18.67%) respondents in Barekese. Regarding assets ownership, 5 (3.33%) respondents in Abuakwa and 7 (4.67%) respondents in Barekese said either they or their partners owned a car whereas 2(1.33%) and 1(0.67%) respondents in Abuakwa and Barekese respectively claimed they or their partners owned a motor cycle.

### Level of Knowledge about EBF in the sub-districts

**Figure 1:**
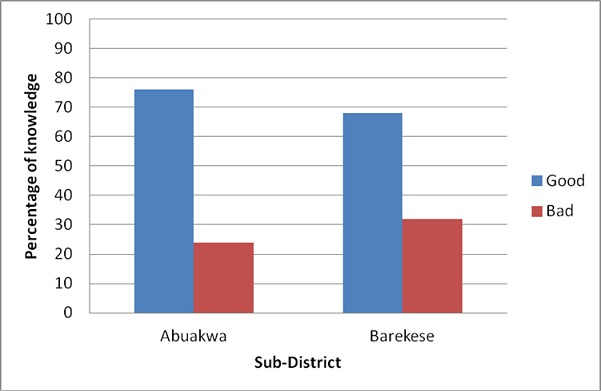
Mothers Level of Knowledge of EBF in the two Sub-districts, 2009

The studied participants demonstrated high level of knowledge of EBF as presented in Figure 1. Table 2 presents details of the cultural reasons for mix-feeding. With regards to colostrum disposal, 70(46.67%) and 50(33.33%) of respondents in Abuakwa and Barekese respectively discarded colostrum regarding it as impure and also as a tradition. However, 80 (53.33%) of respondents in Abuakwa and 100 (66.67%) respondents in Barekese reported that they fed their babies with the yellowish milk that first came out of the breast. There was a significant difference in the two sub-districts with regards to colostrum disposal (P = 0.018).

**Table 2:** Cultural reasons for mix–feeding

	N= 300			
Variable	Community			
	Abuakwa (150)	Barekese (150)	Total	p –value
**Breast milk polluted as result of pregnancy**				
True	69 (46.00)	74 (49.33)	143 (47.67) 157	P = 0.563
False	81 (54.00)	76 (50.67)	(52.33)	
**Colostrum should be discarded**				
True	70(46.67)	50(33.33)	120(40.00)	P = 0.018
False	80(53.33)	100(66.67)	180(70.00)	
**Asiram**				
Yes	35(23.33)	24(16.00)	59(19.67)	P = 0.110
No	115(76.67)	126(84.00)	241(80.33)	
**Fear of mother dying**				
True	36 (24.00)	58 (38.67)	94 (31.33)	P = 0.006
False	114 (76.00)	92 (61.33)	206 (68.67)	

Few mothers in the study areas had their babies suffering from malnutrition-asiram due to improper feeding practices. 35(23.33%) of mothers in Abuakwa and 24 (16%) of respondents in Barekese performed cultural rituals to promote the health of their babies. This was against 115(76.67%) and 126(84%) in Abuakwa and Barekese respectively who did not indulge in the practice. Statistically, there was no significant difference between the two sub-districts (P = 0.110).

The fear of the mother dying and leaving baby behind without it getting used to other foods did not influence their mothers’ decision to mix-feed their babies. There were 26 (24%) and 58 (38.67%) nursing mothers in Abuakwa and Barekese respectively who gave supplementary feeds to their babies for fear of mothers dying early. Contrastingly, 114 (76%) and 92 (61.33%) of respondents in the two areas respectively did not indulge in the practice. Statistically, there was a significant difference between the two sub-districts (P = 0.006).

Majority of the respondents who exclusively breastfed in the two sub-districts were traders, 158 (52.67%). Out of this, 88 (58.67%) came from Abuakwa while 70 (46.67%) were those from the Barekese sub-district. Farmers made up the second category of 78 (26%). Out of this, 22(14.67%) belonged to the Abuakwa division whereas 56(37.33%) consisted of those from Barekese. (Table 3) Almost all the mothers went on maternity leave upon delivery in the two sub-districts. 148(98.67%) of mothers in Abuakwa as well as 147(98%) of mothers in Barekese went on maternity leave. There was no significant difference in the two districts (P = 0.652). One (0.68%) and 5(3.40%) of nursing mothers went on maternity leave for 2 months for Abuakwa and Barekese respectively).

**Table 3:** Occupational and other factors impact on EBF

	N= 300			
Variable	Community			
	Abuakwa	Barekese		
	N (%)	N (%)	Total (%)	P-Value
**Respondent Occupation**				P = < 0.001
Farming	22 (14.67)	56 (37.33)	78 (26.00)	
Trading	88 (58.67)	70 (46.67)	158 (52.67)	
Apprentice	34 (22.67)	23 (15.33)	57 (19.00)	
Civil servant	6 (4.00)	1 (0.67)	7 (2.33)	
**Maternity leave**				
Yes	148 (98.67)	147 (98.00)	295 (98.33)	P = 0.652
No	2 (1.33)	3 (2.00)	5 (1.67)	
**For how long**			N = 295	
One month	1 (0.68)	1 (0.68)	2 (0.68)	P = 0.389
Two months	1 (0.68)	5 (3.40)	6 (2.03)	
Three months	11 (7.43)	13 (8.84)	24 (8.14)	
More than three months	135 (91.22)	128 (87.07)	263(89.15)	
**How often do you go to work**			N = 120	
Twice a week	0 (0.00)	6 (8.70)	6 (5.00)	P = 0.076
Thrice a week	2 (3.92)	6 (8.70)	8 (6.67)	
Five days a week	39 (76.47)	41 (59.42)	80 (66.67)	
Seven days a week	10 (19.61)	16 (23.19)	26 (21.67)	
**Do you carry baby to work**			N = 128	
Yes	37 (67.27)	48 (65.75)	85 (66.41)	P = 0.857
No	18 (32.73)	25 (34.25)	43 (33.59)	
**Who takes care of it**			N = 44	
Mother	8 (40.00)	6 (25.00)	14 (31.82)	P = 0.323
Sister	7 (35.00)	10 (41.67)	17 (38.64)	
Mother-in-law	5 (25.00)	5 (20.83)	10 (22.73)	
Husband	0 (0.00)	3 (12.50)	3 (6.82)	
**Food taken in mother's absence**				
Koko	15(78.95)	24(96.00)	39(88.64)	P = 0.227
Formula	1(5.26)	1(4.00)	2(2.27)	
Expressed milk	1(5.26)	0(0.00)	1(2.27) 2(4.55)	
Others	2(10.53)	0(0.00)		
**How food is stored**			N = 35	
Milk in a fridge	2(12.50)	2(10.53)	4(11.43)	P = 0.722
Koko in a flask	12(75.00)	16(84.21)	28(80.00) 3(8.57)	
Others	2(12.50)	1(5.26)		
**Has baby fallen ill lately**			N = 300	
Yes	59(39.33)	72(48.00)	131(43.67)	P = 0.130
No	91(60.67)	78(52.00)	169(56.33)	
**Diagnoses of baby's illness**			N = 131	
Malaria	23(38.98)	29(40.28)	52(39.69)	P = 0.277
Diarrhea	28(47.46)	37(51.39)	65(49.62)	
Convulsion	0(0.00)	2(2.78)	2(1.53)	
Anaemia	2(3.39)	0(0.00)	2(1.53)	

Source: Field Data 2009.

Almost all the mothers went on maternity leave upon delivery in the two sub-districts. 148(98.67%) of mothers in Abuakwa as well as 147(98%) of mothers in Barekese went on maternity leave. There was no significant difference in the two districts (P = 0.652). One (0.68%) and 5(3.40%) of nursing mothers went on maternity leave for 2 months for Abuakwa and Barekese respectively. Eleven (7.43%) and 13(8.84%) of mothers in the two areas respectively went on leave for three months whereas the majority 135(91.22%) of mothers in Abuakwa and 128(87.07%) of mothers in Barekese went on maternity leave for more than 3 months. There was no significant difference in the two sub-districts (P = 0.389).

For mothers who have resumed work, 39 (76.47%) and 41(59.42%) for Abuakwa and Barekese respectively went to work five days in a week, whereas 10(19.61%) and 16(23.19%) for Abuakwa and Barekese went to work all the days of the week. Statistically, there was no significant difference with regards to length of days spent at work in the two areas under study, (P = 0.076). Regarding food taken by baby in the absence of the mother, (78.95%) and 24(96%) of nursing mothers who have resumed work in Abuakwa and Barekese respectively fed babies below six months with a locally prepared cereal meal. This was against (5.26%) of mothers in Abuakwa who fed babies with baby formula and expressed milk. No nursing mother in Barekese gave expressed milk to baby upon resumption of work. There was no significant difference with regards to supplementary feeds given to baby in the two sub-districts (P = 0.227).

Diarrhea cases were the most prevalent in babies whose mothers had resumed work. The results registered 28(47.40%) and 37(51.39%) for Abuakwa and Barekese respectively. Malaria registered second with 23(38.98) and 29(40.28%) in the two sub-districts. Two (3.39%) of mothers in Abuakwa admitted that their babies suffered from anaemia whereas there was no such case recorded for Barekese. Once again there was no significant difference in the two areas (P = 0.277).

Table 4 presents the findings of community participation in exclusive breastfeeding. Community participation did not register positive results with regards to promotion of EBF. The figures revealed that 108 (72%)and 96 (64%) of nursing in Abuakwa and Barekese respectively were pressured most especially by mothers-in- law to give supplementary feeds. Figures showed no significant difference though, (P = 0.137). Also community members did not seem to play an effective role in EBF promotion in both sub-districts. 143(95.33%) and 133(88.66%) of nursing mothers in Abuakwa and Barekese respectively were rather dissuaded by members of the community. This was against 7(4.67%) and 17(11.33%) from the two areas who admitted they received societal support in relation to the practice of exclusive breastfeeding. This recorded significant difference in the two sub-districts, (P = 0.033). Peer counseling was virtually non-existent in the areas under study. 145(96.67%) of mothers in Abuakwa and 126(84%) of mothers in Barekese did not receive peer counseling on exclusive breastfeeding. This was as against 5(3.33%) and 24(16%) of mothers in Abuakwa and Barekese respectively who claimed the reverse. Statistically, there was a difference in relation to counseling on exclusive breastfeeding in the two areas under study (P = 0.00).

**Table 4:** Community participation

	N= 300			
Variable	Community			
	Abuakwa	Barekese		
	N (%)	N (%)	Total (%)	P-Value
**Pressure to give food**				P = 0.137
Yes	108 (72.00)	96 (64.00)	204(68.00)	
No	42 (28.00)	54 (36.00)	96 (32.00)	
**By whom**		N = 204		P = 0.393
Husband	18 (16.67)	23 (23.96)	41 (20.10)	
Mother-in-law	34 (31.48)	27 (28.12)	61 (29.90)	
Neighbors	29 (26.85)	20 (20.83)	49 (24.02) 41	
Friends	19 (17.59)	22 (22.92)	(20.10)	
Others	8 (7.41)	4 (4.17)	12 (5.88)	
**Encourage to practice E.B.F**		N = 300		P = 0.033
Yes	7(4.67)	17(11.33)	24(8.00)	
No	143(95.33)	133(88.66)	276(92.00)	
**Counseling on E.B.F by volunteers**				P = 0.000
Yes	5(3.33)	24(16.00)	29(9.67)	
No	145(96.67)	126(84.00)	271(90.33)	
**E.B.F practiced by mothers**				P = 0.101
Yes	6(4.00)	13(8.67)	19(6.33)	
No	81(54.00)	66(44.0)	147(49.0)	
Don't know	63(42.00)	71(47.34)	134(44.67)	
**Breast feeding in public**				P = 0.584
Yes	144(96.00)	142(94.67)	286(95.33)	
No	6(4.00)	8(5.33)	14(4.67)	

Source: Field Data 2009

With regards to breastfeeding in public, the results revealed that 144(96%) of nursing mothers in Abuakwa and 142(94.67%) of nursing mothers in Barekese gave breast milk to babies while in public. However, 6 (4%) and 8(5.33%) of mothers in Abuakwa and Barekese respectively did not feel comfortable exposing their breasts in public. There was no significant difference in this notion in the two sub-districts, (P = 0.584).

Two factors were associated with EBF in the univariate logistic model. Unmarried mothers were less likely to practice EBF compared with mothers who were married (OR = 0.46, 95% CI: 0.28, 0.77). Also the duration of breastfeeding was associated with EBF. The adjusted odds ratio was 0.41(95% CI: 0.32, 0.54) in favor of three months compared with six months (Table 5).

**Table 5:** Logistics analysis of factors associated with EBF

Variables	Odds Ratio	P-value	95% CI
**Sub-district**			
Abuakwa	1		
Barekese	0.65	0.06	0.40, 1.02
**Education**			
None	1		
Primary	1.04	0.89	0.56, 1.93
Middle	0.87	0.68	0.45, 1.68
Secondary	0.83	0.69	0.34, 2.03
**Age group**			
15-20	1		
21-30	1.48	0.21	0.80, 2.75
31-40	1.81	0.10	0.90, 3.67
41-50	2.5	0.31	0.42, 14.83
**Marital Status**			
Married	1		
Widowed	0.64	0.52	0.16, 2.57
Single	0.46	0.001	0.28, 0.77
**Nipple pain**			
Yes	1		
No	1.12	0.70	0.63, 0.99
**Ancatta**			
Yes	1		
No	0.67	0.56	0.18, 2.57
**Length of EBF**			
Six months	1		
Three months	0.13	0.001	0.07, 0.24
Twelve months	0.75	0.82	0.07, 8.56
**Mother Occupation**			
Farming	1		
Trading	0.09	0.71	0.52, 1.55
Apprentice	0.97	0.93	0.49, 1.93
Civil servant	2.03	0.41	0.37, 11.32
**Fellow mothers**			
Yes	1		
No	0.68	0.38	0.29, 1.62

## Discussion

This study provides quantitative insights into the determinants of EBF in the Atwima Nwabiagya District of the Ashanti Region of Ghana. The study revealed that mothers’ level of knowledge about EBF was good as such the practice was high in the two sub-districts. It was also found out that traditional cultural practices in the area did not prevent mothers from practicing EBF. All these factors could be attributed to the impact of the Community Based Growth Promotion strategy initiative that was launched in the studied communities in the past.

### The level of knowledge of EBF

Several writers have found a positive correlation between exclusive breastfeeding knowledge and actual practice [11–14]. Though others in the field have seemingly refuted this assertion [15, 16], our study conforms to the former. Mothers who participated in the study had appreciable level of knowledge regarding exclusive breastfeeding and this manifested into actual practice. Mothers demonstrated high knowledge level in relation to what they make of exclusive breastfeeding, duration of exclusive breastfeeding, improper feeding practices and its consequences on health of baby and the recommended feeds for baby after six months. Mothers main source of came from antenatal visits followed by growth promoters in communities. Although Abuakwa (peri-urban) recorded comparatively higher figures with regards to good knowledge of EBF than Barekese (rural), the difference was not significant. Perhaps this may be due to the fact that urban mothers generally possess a high level of literacy hence are better informed especially on novel issues than their rural counterparts. This is consistent with earlier findings on the subject. However, the level of prevalence falls below those presented by the GHS in their (2006) cluster survey report. This can, however, be viewed as an understandable phenomenon since the cluster survey might have made use of a larger sample size than the current study.

### The cultural practices associated with EBF

The cultural practices of the people in relation to breastfeeding did not affect mothers’ decision to exclusively breastfeed their babies in the two sub-districts. Cultural myths and practices associated with breastfeeding have been a bane to the practice of Exclusive Breastfeeding especially in Africa [11, 17, 18]. Ranging from colostrum disposal through to herbal medications and the eventual mix-feeding syndrome, mothers have been confronted with the challenge of how best to care for their babies. Fortunately nursing mothers in the Atwima Nwabiagya district have transcended these cultural stalemates and are preoccupied in ensuring that their babies benefit solely from essential nutrients expelled in breast milk for the first six months of life, although this does not go without hitches. Results indicate that several mothers still indulge in mix feeding for fear of their early demise. Relatedly figures representing mothers who discarded colostrum are significant enough to warrant attention though less than those found out in rural Tanzania [19 and the West Bengal State of India [20].

Sexual prohibition during breastfeeding is a taboo held by many African countries [18] with Ghana non-exempt. The assertion that mothers’ milk is polluted when she begins sexual union with her partner has compelled many mothers to introduce complementary feeds early in baby's life. Inherent in the taboo is the rationale to postpone childbirth, prevent unintended pregnancy and to afford mothers enough time to nurse their babies. However, with post-natal counseling on family planning methods coupled with the erstwhile vibrant activities of growth promoters, this traditional adherence is fast losing its grip on the people. These cultural issues influencing EBF have been reported elsewhere [18].

### Farming and exclusive breastfeeding

Farming as a profession was cited as a major setback to the practice of EBF in the Barekese sub-district. Though majority of the respondents were traders, there were more traders in Abuakwa than Barekese. Since more women in Abuakwa exclusively breastfed their babies than Barekese it is realistic to assume a positive relationship between the trading profession and exclusive breastfeeding. Mothers who trade their wares either within the neighborhoods or in the market carry their babies along and periodically breastfeed. Female farmers from rural Ghana leave babies in the care of others and travel miles away to attend to farming activities. The main food fed to baby in the absence of the mother is a locally prepared cereal meal, tenaciously increasing baby's chances of contracting infection.

Most mothers worldwide enjoy a rest period following birth though the length of time differs with respects to mothers’ occupation and the organization she works for. The period spans through one week to three months. The longer the period, the greater the chances of practicing exclusive breastfeeding and vice versa. The leave period provides mothers the opportunity to be closer to their babies and to provide the best possible care. Since mother and baby are always together, the likelihood of practicing EBF is high. The opposite holds true [21, 22]. More mothers in Abuakwa, than Barekese went on maternity leave for more than three a month's period. This revelation is in consonance with the findings elsewhere in England [21]. Again, the rural sub-district (Barekese) recorded a high number of babies suffering from diarrhea. However, babies in both areas who were not subjected to mix feeds did not report of any diarrhea disease. This outcome supports the findings reported by Kosmala-Anderson et al [21]. A confirmation of the fact that babies who breastfed exclusively for the six months are less likely to suffer from constipation or diarrhea and are also less prone to childhood diseases including juvenile diabetes, allergies, asthma, eczema, gastrointestinal, urinary and respiratory tract infections.

### Absence of community volunteers

The virtual absence of community volunteers in exclusive breastfeeding promotion became the utmost disincentive to the practice of EBF in the two areas. The timing of the study coincided with the period that witnessed the fold-up of growth promotion activities. Until then mothers had benefitted immensely from counseling and support on exclusive breastfeeding from growth promoters. Having lost touch with these activities exposed mothers to the vagaries of society to mix-feed. Mothers- in- law are a force to reckon with in this regard. Their conservative approach to issues has made many a change a daunting task. This possible relapse could be due to the fact that growth promotion initiative had only lasted four years in the district and definitely not enough a period to change a societal issue. Elsewhere initial and recurrent contacts with peer counselors were associated with a significant increase in exclusive breastfeeding initiation, duration and rates [23–26].

Breastfeeding, to mothers in this Sub-region, is a natural phenomenon and mothers do not hesitate to breastfeed wherever they find themselves. This contrasts with what pertains in the Western world [15]. What should, however, be of worry is whether mothers’ hands and nipples are cleaned under the circumstances to breastfeed and what recommendation is in place to ensure that in their bid to breastfeed at any time and place that baby demands breast milk, mothers do not end up endangering the lives of babies though infection..

### Study limitations

The vast nature of the district made it difficult for the researchers to cover the entire district. Nonetheless, this might not affect the results since those who eventually took part in this study were randomly selected. Financial constraint was a major determinant of the sample size.

## Conclusion

The level of knowledge of EBF in the two sub-districts was generally good and as such the practice was high. The cultural practices of the people in relation to breastfeeding did not affect mother's decision to exclusively breastfeed their babies. We also noticed that mothers who were farmers were less likely to practice EBF compared with other professions like trading. However, the absence of community participation in all aspects pertaining to EBF initiatives became the utmost barrier to the practice of EBF within the study area. The District Health Administration should ensure that EBF campaigns through outreach clinics are enhanced by addressing, particularly, mothers’ occupational needs and effective EBF practices. Also, the Ghana Health Service should consider reinstating the CBGP intervention programme while adequately motivating growth promoters. Locally, the Assembly could initiate an award scheme to appreciate mothers who exclusively breastfeed. This could serve as incentives to get mothers adopt the practice.
